# Tight junctions in pulmonary epithelia during lung inflammation

**DOI:** 10.1007/s00424-016-1917-3

**Published:** 2016-12-05

**Authors:** Oliver H. Wittekindt

**Affiliations:** Institute of General Physiology, Ulm University, Albert-Einstein-Allee 11, 89081 Ulm, Germany

**Keywords:** Lung, Inflammation, Asthma, COPD, ARDS, Tight junctions

## Abstract

Inflammatory lung diseases like asthma bronchiale, chronic obstructive pulmonary disease and allergic airway inflammation are widespread public diseases that constitute an enormous burden to the health systems. Mainly classified as inflammatory diseases, the treatment focuses on strategies interfering with local inflammatory responses by the immune system. Inflammatory lung diseases predispose patients to severe lung failures like alveolar oedema, respiratory distress syndrome and acute lung injury. These life-threatening syndromes are caused by increased permeability of the alveolar and airway epithelium and exudate formation. However, the mechanism underlying epithelium barrier breakdown in the lung during inflammation is elusive. This review emphasises the role of the tight junction of the airway epithelium as the predominating structure conferring epithelial tightness and preventing exudate formation and the impact of inflammatory perturbations on their function.

## Introduction

The surface of the airways and the alveoli is shielded by an epithelial cell layer. This epithelium forms the first defence line against airborne noxae and prevents invasion of the organism by infectious particles. It also traps airborne particulate matter and removes them from the airways. Furthermore, it senses perturbations and orchestrates the immune response [27].

Inflammatory lung diseases form a heterogeneous disease entity, which subsumes infectious lung diseases, allergic responses, asthma and chronic obstructive pulmonary disease (COPD). They significantly increase susceptibility to lung injury and respiratory distress syndrome [135]. The breakdown of the epithelial barrier is a hallmark in respiratory distress syndromes and can be identified via the appearance of high molecular weight serum proteins in broncho-alveolar lavage from patients [50].

The barrier function of the lung epithelium depends on so-called tight junctions (TJ). These heteromeric protein complexes form the sealing interface between adjacent epithelial cells [109]. The damage of TJ is the major cause of epithelial barrier breakdown during lung inflammation. Even though breakdown of lung epithelial barrier is life threatening, TJs of the lung epithelium and their regulation/disturbance in health and disease are less elaborated.

## Organisation of the lung epithelium

**Fig. 1:**
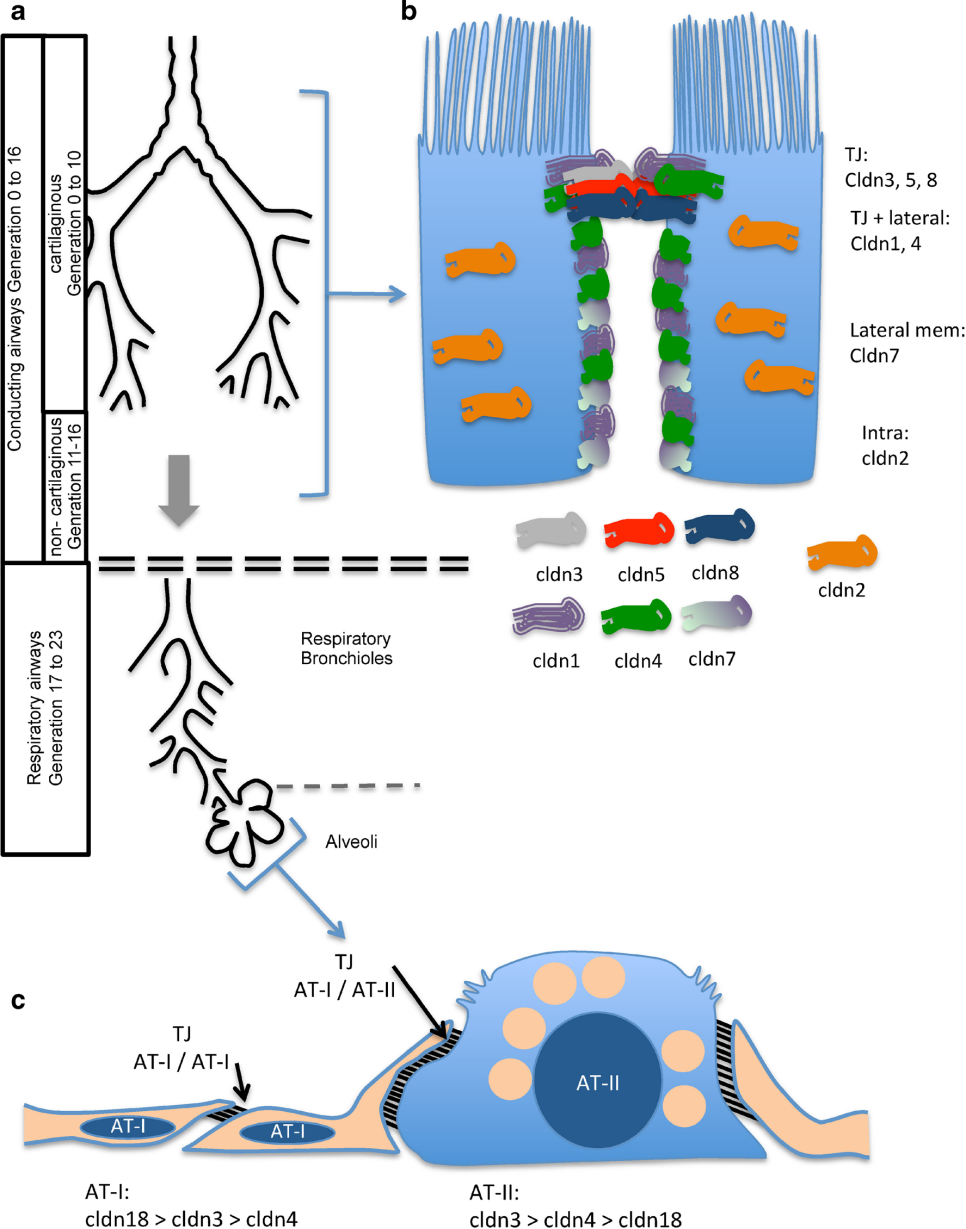
Organisation of the airways and the airway epithelium. **a** The airways are subdivided into conductive and respiratory sections. The conductive airways contain cartilaginous and non-cartilaginous airways. The respiratory section constitutes the respiratory airways and the alveoli. **b** Scheme gives an overview of intracellular claudin (cldn) distribution in airway epithelial cells. The claudins predominantly localised at the tight junctions (TJ) (cldn3, 5, 8), localised at the tight junctions and the lateral membrane (cldn1, 4), predominantly localised basolateral from the TJ (cldn7) and localised intracellular (cldn2) are depicted. **c** Scheme of the alveolar epithelium. The alveolar epithelium constitutes alveolar type I (AT-I) and type II (AT-II) cells. The tight junctions between adjacent AT-I cells are narrower than those between AT-I and AT-II cells. The most abundantly expressed claudins in AT-I and AT-II cells are cldn3, 4 and 18. Their abundance sequences for each cell type are given below

The airways can be subdivided into a conducting and a respiratory region. The conducting airways comprise the cartilaginous airways from the trachea to the 10th generation of the bronchial tree, and the non-cartilaginous airways of the small bronchi to the terminal bronchioles until the 16th generation. Generations 17 to 23 are considered as respiratory airways, which finally end in the alveoli (Fig. 1a). The conducting airways ensure the humidification of inhaled air, sensing of irritants, trapping of inhaled particulate noxae and their removal from the surface of airways by mucocilliary clearance. The airways are lined by a pseudo-stratified columnar ciliated epithelium. The epithelia of the cartilaginous airways are composed of glands, ciliated cells and mucus-producing goblet cells with the number of glands and goblet cells decreasing and the number of mucus-producing club cells increasing from proximal to distal. In the non-cartilaginous airways, neither glands nor goblet cells are present, but an increasing number of columnar epithelial cells and club cells are found. The respiratory airways form a transition between the conducting part and the alveoli. They guide the inhaled air towards the alveoli and contribute to the gas exchange. They are lined by a non-ciliated epithelium, which is distinct from the conducting airway as well as from the alveolar epithelium. However, with respect to its architecture, it is more related to the conducting airways than to the epithelium, which lines the alveolar space. Within the respiratory section, mucus-producing cells are sparse and are completely absent as closer the epithelium is localized to the alveolus. The alveolar epithelium compromises only two types of cells, alveolar type I and type II cells. Its architecture optimises it for gas exchange.

## The epithelium as a barrier between compartments

The epithelium of the conducting and respiratory airways as well as the epithelium of the alveoli constitutes a barrier that separates the air-filled compartment of the respiratory system from the aqueous interstitial compartment. This separation of both compartments from each other is a major task of the airway epithelium; yet, at the same time, the epithelium also has to manage a regulated exchange of solutes and water between these compartments. Two main transport pathways across the epithelium—trans- and paracellularly—are well established. The transcellular transport pathway depends on the polarised distribution of ion channels and transporters localised in the apical and basolateral membrane. Upon resorption, Na^+^ enters the cell via apically localised epithelial sodium channels and is released into the interstitium via basolaterally localised Na^+^/K^+^-ATPase. Paracellular transport runs through the extracellular compartment between the lateral membranes of neighbouring epithelial cells. It depends on diffusion processes, which are driven by chemical and electrochemical gradients across the epithelial cell layer. This paracellular transport is controlled by heteromeric protein complexes, which are formed at cell-cell interfaces at the apical side of the lateral membranes between adjacent cells. These complexes are called tight junctions (TJ) and seal the lateral space at its apical side. Despite the importance of sealing the epithelium, TJs must confer a certain permeability to ensure a sufficient exchange and transport across the epithelium. The permeability of TJs depends on the protein composition and can be adjusted to be permeable for solutes of different charges, sizes and water [45, 70]. TJs are composed of occludin and various claudins with the claudin composition being the main regulator of tight junction permeability (permselectivity) [71, 109]. Claudins are proteins with four membrane spanning domains (tetraspanins) and constitute a unique protein family consisting of 27 members [42]. A loss of TJ permselectivity in the airways results in an un-controlled leakage of high molecular weight proteins and water into the airways, which finally results in the formation of alveolar oedema and respiratory distress syndrome.

Although knock-down of individual claudins in mice so far revealed rather mild lung phenotypes [30, 64, 75, 78, 126], implicating that the lung can compensate the loss of claudin function to a certain extent, some knockout strains developed an increased susceptibility to acute lung injury [64, 75, 78] indicating the importance of claudins and TJ function as a risk factor in developing acute lung injury and respiratory distress syndrome.

## Tight junctions of airway epithelia

The epithelia of the conducting and respiratory airways are optimized to maintain its specific functions and so is the claudin composition of the TJ (Fig. 1b). Immunohistological experiments revealed that the epithelia of cartilaginous and non-cartilaginous airways is positive for cldn1, cldn2, cldn3, cldn4, cldn5, cldn7 and cldn8 [24, 62, 63, 66]. However, the intracellular localisation of these claudins differs. Cldn2 is localised in intracellular stores rather than in apico-lateral TJ complexes [62]. In contrast, cldn3, cldn5 and cldn8 were detected exclusively in TJ complexes [24, 66], and cldn1 and cldn4 localise throughout the lateral membranes as well as to the apico-lateral TJ complexes [24]. Cldn7 localises at the lateral membranes basolateral of the TJ complex [24].

## Tight junctions of the alveolar epithelium

The alveoli form sacks at the most distal parts of the airways. They are lined by the alveolar epithelium (Fig. 1c), which is part of the diffusion barrier across which the gas exchange occurs. To this end, the main surface of the alveolar epithelium forms an extremely thin cell layer with a unique architecture [26, 138]. Two cell types constitute the alveolar epithelium, the squamous alveolar type I (AT-I) cells and the cuboidal alveolar type II (AT-II) cells. The lateral contact between adjacent AT-I cells is sealed by a narrow band formed by TJ complexes. In contrast, the lateral contact between adjacent AT-I and AT-II cells is formed and sealed by a much broader TJ complex [138]. The cell type specificity of TJ morphology is reflected by heterogeneity of claudin expression in alveolar epithelial cells. Both alveolar epithelial cell types express cldn3, cldn4 and the splice variant cldn18-1 most abundantly [36, 74, 132]. Their phylogeny places cldn3 and cldn4 into the class of classical claudins, whereas cldn18 is a member of the so-called non-classical claudin family [72]. However, the claudin expression pattern differs between AT-I and AT-II cells. Cldn18 transcripts account for 56%, cldn3 transcripts for 31% and cldn4 transcripts for 10% of all claudin transcripts in AT-I cells. AT-II cells exhibit a different quantitative sequence. In this cell type, 67% of all claudin transcripts are cldn3 transcripts, 23% are cldn4 transcripts and only 7% of the claudin transcripts encode for cldn18 [74]. These claudins are all elevated in bronchio-alveolar lavage 24 h after acute lung injury [61] underscoring their dominant expression especially within the alveolar epithelium.

## Caludins of the alveolar and airway epithelium

### Cldn3

Cldn3 modifies paracellular permeability upon oxidative stress in gastric epithelia [44] and upon exposure to the inflammatory factors TNF-α in submandibular glands [88]. Cldn3 was demonstrated to reduce paracellular permeability, when overexpressed in Madin-Darby canin kidney cells [90]. Based on these studies, cldn3 can be accounted to the group of sealing claudins. However, in cultivated alveolar epithelial cells, cldn3 increases paracellular permeability and opposes the sealing effect of cldn4 [91].

### Cldn4

Overexpression studies revealed cldn4 as a claudin that decreases paracellular permeability [22, 89, 145]. A sealing function of cldn4 was also demonstrated in cldn4 knockout mice (64). These mice demonstrated an increased susceptibility to hypoxia and ventilator induced lung injury and an increased solute permeability of the alveolar epithelium without altering its transepithelial electrical resistance [64]. During early stages of acute lung injury, cldn4 becomes up-regulated, possibly to limit lung oedema formation [144]. A role of cldn4 in compensatory alveolar fluid clearance is further supported by the observation that increased cldn4 protein levels are associated with increased alveolar water resorption [105]. Hence, cldn4 has sealing function in the alveolar epithelium that is necessary for volume homeostasis of the alveolar liquid layer.

### Cldn18

Four different splice variants were identified for murine cldn18. The variants cldn18-1 and cldn18-2 are generated by alternative splicing of the first coding exon. Alternative splicing of the fourth and fifth coding exon results in the variants cldn18-1.1 and cldn18-1.2 as well as in cldn18-2.1 and cldn18-2.2. Cldn18-1 variants are predominantly expressed in the lung whereas cldn18-2 variant expression is found predominantly in the stomach [94]. Cldn18 knock-down mice showed a fairly mild phenotype. Cldn18 knockout disturbs TJ formation in the alveolar epithelium which is in line with an increase in paracellular permeability [75, 78]. Despite disturbed barrier function, alveolar liquid volume homeostasis was stable and susceptibility to ventilator-induced lung injury was even reduced in knockout animals. These mild effects are possibly due to increased water and ion transport capacities and a compensatory elevation of cldn4 expression levels [78]. Therefore, cldn18-1 plays a role in TJ organisation and may also have a sealing function in alveolar epithelia.

### Cldn1

Cldn1 is ubiquitously expressed along the airway epithelium [62, 63]. It confers sealing properties to TJ [24, 56, 87]. Phosphorylation at its N-terminal domain by MAP kinases enhances cldn1’s sealing properties [37]. Cldn1 does not localise exclusively at the TJ but throughout the lateral membranes [24, 62], which hints that cldn1 may also regulate cell-cell attachment between adjacent epithelial cells. This agrees with the observation that cldn1 supresses tumour invasion and metastasis [19] and is involved in modulating migration of A549 cells [111].

In accordance with the sealing properties of cldn1, interfering with cldn1 abundance decreases tightness of airway epithelia. Protein kinase D3 impairs epithelial barrier function in airway epithelia via cldn1 down-regulation [17]. Activation of protease-activated receptor 2 (PAR2) transiently down-regulates cldn1 expression and decreases epithelial permeability with a similar time course [95]. In contrast, thymic stromal lymphopoietin (TSLP) [85] and peroxisome prolifertator-activated receptor (PPARγ) [96] both increase cldn1 expression and improve tightness of human nasal epithelia.

### Cldn2

Cldn2 introduces a high permeability for cations into TJ [8, 38, 57, 149]. It is the only claudin described so far that also forms paracellular pores for water [106, 141]. The pathway of water flux across airway epithelia is a matter of debate. Studies by the Verkman group, employing genetic knock-down of aquaporins, revealed a minor contribution of the transcellular, and therefore TJ independent pathway, on transepithelial water transport in the lung [83, 84, 114, 130, 131], suggesting a major contribution of TJ-dependent paracellular water flux to overall transepithelial water flux. However, other studies found that perturbations of aquaporin activity disturb transepithelial water transport and volume homeostasis in the airways [2, 32, 35, 113, 127], suggesting that TJ-independent water transport pathways through aquaporins contribute significantly to fluid transport across the airway epithelium. A more recent study now suggests that both processes occur in a different manner, where basal water transport activity is dominated by a paracellular pathway, whereas a compensatively increased water resorption is predominantly carried by an aquaporin-dependent transcellular pathway [110]. In airway epithelial cells, cldn2 localises in intracellular stores rather than at the TJs [62] and is regulated by TNF-α [86]. However, its specific role in airway epithelial cells remains elusive.

### Cldn5

Cldn5 expression depends on lung developmental stage. During the canalicular stage, alveolar epithelial cells express cldn5 [63], which agrees with cldn5 expression of primary cultivated foetal alveolar cells [28]. The healthy alveolar epithelium expresses low levels of cldn5 [74]. The airway epithelium expresses cldn5 independently of developmental stage [24, 62, 63]. Paracellular epithelial permeability in lung epithelia increases with increasing cldn5 expression [24, 34, 132, 134]. This is accompanied by an increased susceptibility to oedema formation and lung injury as it was observed in lung for patients and lung epithelia after chronic alcohol ingestion [34, 41, 112].

NFκB is a major regulator of cldn5 in the lung. Inhibition of basal NFκB activity in airway epithelial cells increases cldn5 expression in the absence of inflammation [134]. This is in line with the observation that TNF-α, which activates the classical NFκB signalling pathway, down-regulates cldn5 promotor activity [11, 18]. Indeed, increased TNF-α levels attenuate cldn5 expression in a mouse model of acute lung inflammation [86]. Overall, NFκB-dependent reduction of cldn5 seems to improve epithelial barrier function during lung inflammation and seems to be beneficial with respect to epithelial function. Contrary to that, virus infection-induced lung injury is associated with decreased cldn5 expression levels [10, 54, 79]. In the lung, cldn5 expression is also observed in endothelial cells of blood vessels [63], and Cldn5 is demonstrated to protect endothelial barriers from LPS-induced leakage.

It is not yet clear whether this dualism of cldn5 function in the lung is due to different effects of cldn5 on endothelial and epithelial cell-cell interfaces, due to different regulatory mechanisms involved or simply due to differences in the underlying damage. However, because of this dualism, it is difficult to judge which effect dominates in the lung during inflammation.

### Cldn7

IFN-γ enhances cldn7 expression and the transepithelial electrical resistance in submandibular glands [1]. This highlights cldn7 as a sealing claudin at first sight. However, studies addressing the permeability properties of cldn7 revealed a more complex function of cldn7 on TJ permeability. Overexpressing cldn7 in the porcine kidney, epithelial cell line LLC-PK1 resulted in an increased transepithelial electrical resistance via reducing paracellular Cl^−^ permeability while forming a Na^+^ pore. While cldn7 confers ion or charge selective pores to the TJ and reduces the overall permeability for ions, it increases the permeability of TJ to uncharged molecules [6]. Silencing of cldn7 expression in LLC-PK1 reduces transepithelial resistance and increases paracellular permselectivity for Na^+^ over Cl^−^ [51]. These results agree with the results from the above-cited cldn7 overexpression experiments [6]. However, when cldn7 was silenced in Madin-Darby canine kidney cells (MDCK), the transepithelial resistance decreased and paracellular permselectivity for Cl^−^ increased over that for Na^+^ [51]. This indicates that the effect of cldn7 on TJs depends on its cellular background. Phosphorylation of cldn7 within its C-terminal, intracellular domain via WNK4 kinase modulates cldn7 permeability. It instead promotes paracellular ion permeability and increases Cl^−^ permselectivity of TJ [124]. Possibly, differences in post-transcriptional protein modification explain the variability in cldn7 function observed within different cell types.

No lung phenotype is described for cldn7 knockout mice so far [30, 125]. However, cldn7 knockout resulted in renal salt wasting and chronic dehydration [125] which underscores the pivotal role of cldn7 in transepithelial ion transport.

Cldn7 knockout affects expression of its lateral adhesion complexes by down-regulation of the epithelial adhesion molecule EpCAM [73] in case of intestine-specific inducible knockout strains [122] or in case of non-organ specific knockout strains via down-regulation of integrin-α2 [30]. Only in the later case, the mucosal architecture of intestinal epithelium was massively disturbed due to cldn7 knockout [30]. In airway epithelia, cldn7 localises throughout the lateral membrane of epithelial cells [24]. Possibly, organisation of lateral adhesion junctions between epithelial cells is one of the major tasks of cldn7 in the airways. This hypothesis is strengthened by the observation that cldn7 regulates cell attachment by interacting with integrin-β1 in human lung cancer cells [82].

### Cldn8

Cldn8 augments tightness of TJ [9, 60, 150] by selectively reducing paracellular permeability to monovalent and divalent cations [150] as well as to protons, ammonium ions and bicarbonate [150], whereas Cl^−^ permeability of TJ remained unaffected. Therefore, cldn8 increases permselectivity of TJ for Cl^−^. Cldn8 interacts with cldn4, and cldn8 is required to localise cldn4 at the TJs in MDCK cells [52]. This led to the conclusion that both claudins are required for paracellular Cl^−^ permselectivity. Cldn8 mediated sealing of TJ to Na^+^ parallels Na^+^ absorption in human colon cells [52], and hence, it is proposed that cldn8 augments sodium resorption by preventing paracellular leakage of Na^+^. Investigation of cldn8 function in the lung is rather sparse. Immnunohistochemical experiments revealed that cldn8 localises along conductive and respiratory airway epithelia, where it accumulates apico-laterally at the TJs [66]. In the alveolar epithelium, cldn8 staining revealed a faint and cytoplasmic staining in some alveolar type II cells [66]. In the airway epithelium, cldn8 is up-regulated by glucocorticoids but not by mineralocorticoids [66]. It is required for recruitment of occludin to the TJs, and thereby, it confers sealing properties and Cl^−^ permselectivity to the TJ [66].

## The airway epithelium during inflammation

**Fig. 2:**
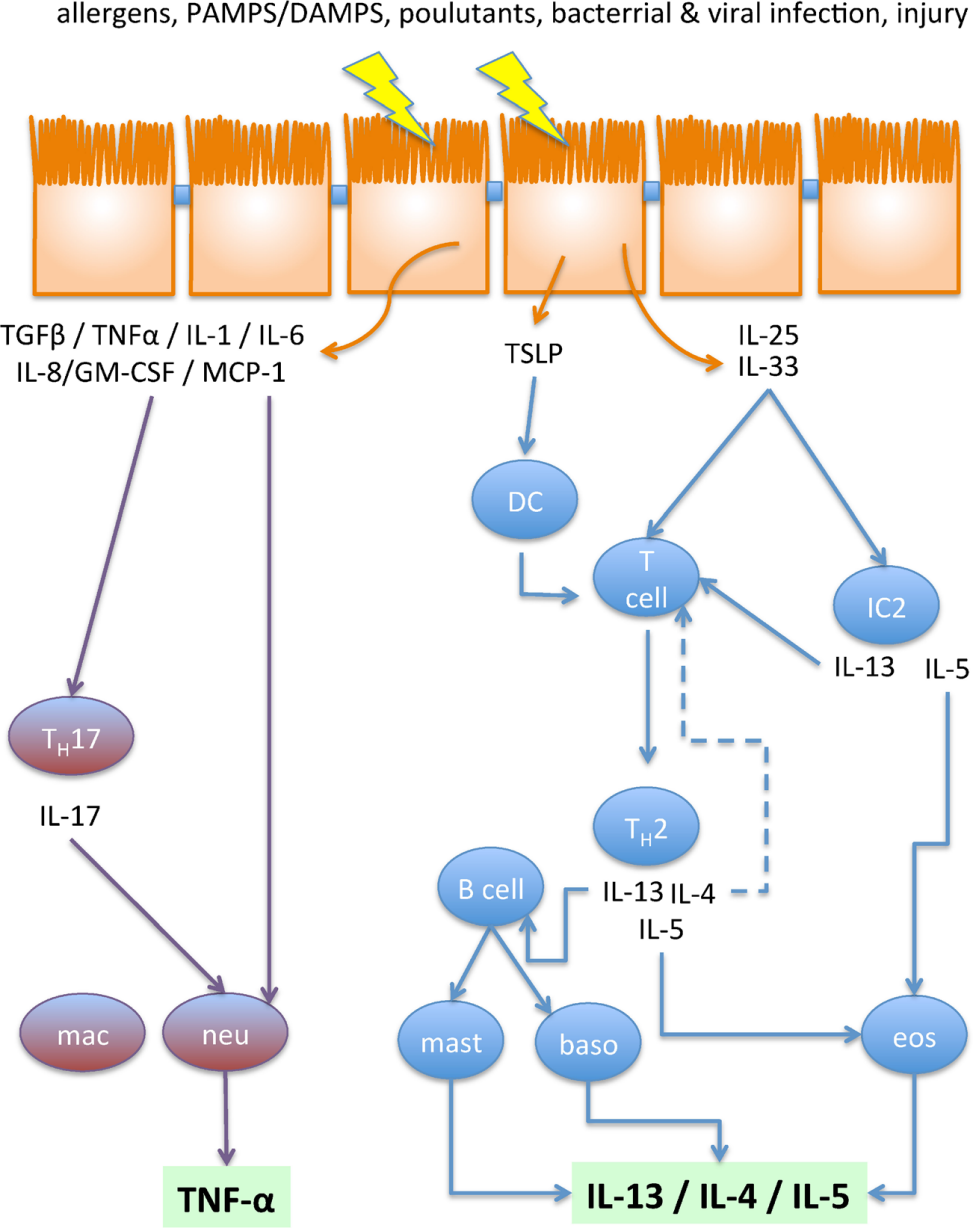
Overview of the dominating immune response activating pathways. Perturbations of the airway epithelium initiate the release of cytokins directly from epithelial cells. TSLP, IL-25 and IL-33 activate T helper cell type 2 (T_H_2)-driven inflammation, which is dominated by eosinophils (eos). This response results in an enrichment of IL-4, IL-13 and IL-5. Especially IL-13 and IL-4 are the dominating factors, which damage the epithelium during T_H_2-driven inflammation. Neutrophil response dominates the T_H_2-low inflammation. It becomes activated via neutrophil recruitment by cytokines either directly released from epithelial cells or indirectly via activation of T helper cell type 17 (T_H_17) cells. *IC2* innate lymphoid cell type 2, *baso* basophil, *DC* dendritic cell, *mac* macrophage, *mast* mast cell, *neu* neutrophil

The airway epithelium constitutes the first cell layer that gets into contact with inhaled noxae and has to impede impending injuries. However, it also comprises an ideal structure to sense inhaled noxae (Fig. 2). Human bronchial epithelial cells were recently identified as a source of cytokines in the lung, and therefore, the airway epithelium was proposed as a sensor of airborne noxae [27]. Bacterial and viral infections induce TNF-α, IL-1α, IL-1β, IL-6, IL-8 and IL-18 [21, 77, 101, 115, 143]. In addition, allergic agents such like cationic peptides [21], proteolytic active [12, 29, 58, 59, 68, 117] as well as non-proteolytic allergens [98] induce the release of IL-6, IL-8, granulocyte macrophage colony-stimulating factor (GM-CSF) and monocyte chemotractant protein 1 (MCP-1). The response to proteolytically active allergens involves store-operated Ca^2+^ entry in epithelial cells [58, 59], and it should be noted that bacterial exotoxins also activate store-operated Ca^2+^ entry [128]. Other factors, which belong to danger-associated molecular patterns (DAMP), like adenosine [117], prostaglandin [20] or histamine [120], initiate IL-1β, IL-6, IL-8 and GM-CSF production and release. Further, stimuli for chemokine release from airway epithelial cells are inhaled air pollutants [47, 119] and cold [108]. More recent investigations revealed airway epithelial cells as a source of IL-25, IL-33 and thymic stromal lymphopoieitin (TSLP). This subset of cytokines is released by airway epithelial cells upon viral [15], bacterial [33] and fungal infection [48] as well as a result of allergen stimulation [23, 53, 65, 93, 97, 102].

These epithelial responses are important for recruiting immune cells and orchestrating their complex interaction at the side of airway perturbation. A balanced inflammatory response is a requisite to successfully protect lung from damage. However, damage of tissue depends on an overwhelming inflammatory response.

## Tight junctions and asthma

Asthma is a complex disorder, which involves environmental interactions and chronic inflammation of the airways. According to its immunology, asthma can be subdivided into two major types, the T helper type 2 cells high (T_H_2-high) and the T helper type 2 cells low (T_H_2-low) endotype [116].

T_H_2-high endotype is initiated directly via IL-25 and IL-33 released by epithelial cells or indirectly via stimulation of innate lymphocytes type 2 [80]. A third initiation pathway acts via TSLP stimulation of dendritic cells, which attenuate T_H_2 polarisation. Neither TSLP, IL33 nor IL23 impairs airway epithelial barrier [107]. T_H_2 and ILC2 cells recruit eosinophils, and, via induction of B cells, also mast cells and basophils. Thus, T_H_2-high endotype is characterised by enrichment of eosinophils, basophils and mast cells [104]. The accumulation of these immune cells results in a typical chemokine pattern, called T_H_2-pattern, with high levels of IL-4, IL-5 and IL-13.

T_H_2-low endotype is initiated via IL-1β, TGF-β and IL6, which induce recruitment of neutrophils via stimulation of IL-17 release from T helper cells type 17 (T_H_17). An enrichment of neutrophils characterises this asthma endotype [104] (Fig. 2).

Especially, the TH2-high endotype is involved in exacerbations and also suggested to increase epithelial damage in asthma patients.

### T_H_2-driven TJ damage: IL-4/IL13

IL-4 and IL-13 directly interfere with TJ. In Calu3 cells, IL-4 induces disassembly of TJ molecules [100], IL-4 and IL-13 increase paracellular permeability in human bronchial epithelial cells [107, 118], in sinosonal epithelial cells [142] and in air-liquid interface cultivated paranasal sinus mucosa cells [16]. Although IL-4 and IL-13 show similar effects on TJ in these airway epithelial cell models, depending on the investigated model, they act via different pathways. In Calu3 epithelia, IL-4 is reported to activate an EGFR-dependent MAPK/ERK1/2 pathway [100], whereas in human bronchial cell derived epithelia IL-4 as well as IL-13 act via the Janus kinase/signal transducer and activator of transcription (JAK/STAT) pathway [107], most likely via binding to the same receptor, namely heteromeric IL-13Rα/IL-4Rα receptor [139]. Indeed, both receptor subunits have an overlapping expression pattern in lung epithelia. IL-4Rα as well as IL-13Rα both were detected in human bronchial epithelial cells in vitro and in vivo [4, 46, 129, 133, 139]. IL-13Rα expression levels are increased in bronchial biopsy specimens from asthma patients which probably confers increased bronchial sensitivity to T_H_2 cytokines [69].

The effect of IL-4 and IL-13 on claudin expression pattern differs between the investigated epithelia. Epithelial brush samples from asthma patients with high IL-13 levels and IL-13-exposed human bronchial epithelial cells showed decreased cldn18.1 levels [118]. In mouse, lung IL-13 reduces cldn18 while increasing cldn4 expression [118]. In another human bronchial epithelial cell model, IL-13 and IL-4 are reported to reduce protein density at the TJ without causing major changes in cldn1, cldn2, cldn3 and occludin protein levels [107]. Sinonasal epithelia from patients with allergic fungal rhino sinusitis, which display high T_H_2 cytokine levels, showed increased levels of cldn2 which most likely contributes to the tight junction leakiness [16]. Overall, IL-4 and IL-13 have high potential to damage TJ in airway epithelia.

## TJs in early lung inflammation, COPD and acute lung injury

Neutrophil enrichment in lung is a hallmark of early lung inflammation, COPD and acute lung injury. Elevated levels of neutrophils are a major criterion to distinguish COPD from asthma [3]. The recruitment of neutrophils is driven via direct perturbation of epithelial cells. Airborne insults such like cigarette smoke, diesel dusts, bacterial infection and allergens induce the release of chemokines IL6, IL8, TNF, IL-1, GM-SCF, MCP-1 from airway epithelial cells [12, 20, 21, 29, 47, 58, 59, 68, 77, 98, 101, 115, 117, 119, 120, 143]. Release of these cytokines either recruit neutrophils directly [140] or induce IL-17 release from T_H_17 cells [137]. The outcome of neutrophil enrichment is a cytokine profile, which is dominated by TNF-α, IL-1β, IL-6, IL-17, IL-18, IL-32 and TGF-β [14] (Fig. 2).

Especially, TNF-α plays a major role in perturbing tight junctions in airway epithelia. TNF-α acts via NFκB, which is considered as a major regulator of tissue inflammation [99]. In mammals, the NFκB family consists of five transcription factors, p50, p52, REL, RELA and RELB, which form either hetero- or homodimers. In the resting stage, NFκB dimers bind proteins of the NFκB inhibitor family IκB. Activation of NFκB signalling induces expression and release of pro-inflammatory factors such like IL-1, IL-2, IL-6, IL-8 G-CSF, GM-CSF, TNF-α, TNFβ and IFN-β [40]. This pro-inflammatory effect of NFκB is also demonstrated for airway epithelial cells [43, 67, 103, 121, 147, 148]. Activation of NFκB by TNF-α follows the canonical or classical activation pathway, which involves NEMO, IKKα and IKKβ mediated phosphorylation of IκB and its subsequent dissociation from the p50 complex [99]. The activation of the canonical or classical NFκB pathway increases paracellular permeability for instance in intestinal barrier [5] and in retinal endothelia [13]. Also, in airway epithelia, TNF-α down-regulates paracellular epithelial barrier function [25]. The genetic modulation of TNF-α in mice revealed that TNF-α negatively regulates tight junction proteins, namely cldn2, cldn4, cldn5 and ZO-1 in the lung, which results in increased alveolar permeability [86]. Activation of NFκB also induces a pro-inflammatory response, and thus, it induces the production and release of a variety of proinflammatory factors, which potentially interferes with TJ integrity. However, NFκB has the ability to modulate TJ permeability directly. Even in the absence of inflammation, inhibition of constitutive basal NFκB activity in human airway epithelial cells causes an up-regulation of cldn5 expression, disturbs TJ organisation and increases paracellular permeabilty [134]. Furthermore, neutralising antibodies for IL-6 and IL-8 did not hamper TNF-α-induced perturbations of TJs in human airway epithelial cells [43]. Thus, TNF-α has the potency to perturb TJ function directly via NFκB without further need of any para- or autocrine mechanisms.

Interfering with the transcriptional control of TJ proteins is not the only pathway involved in TNF-α-mediated disruption of TJs. TNF-α also interferes with intracellular localisation of TJ proteins, even of those which expressional level remains unaffected [25]. In human colon epithelial cells, TNF-α induces disassembly of TJ supposedly via src-kinase mediated modulation of protein turnover at the TJ [7]. Also, in human airway epithelial cells, src-kinase inhibition attenuates TNF-α-induced TJ disruption and restores at least partly intracellular localisation of TJ proteins [43].

The pivotal role of the TNF-α/NFκB pathway on lung epithelial barrier function is underscored by the fact that inhibition of the signalling pathway reduces the risk of acute lung injury in models of inflammatory lung diseases [146, 151, 152].

## TJs and respiratory failure

Breakdown of airway epithelial barrier function is a diagnostic marker for respiratory distress syndrome and lung injury [50]. It indicates TJ breakdown to be causative in developing this life-threatening lung failure. Inflammation is a major factor, which predisposes patients to lung injury and respiratory distress syndrome [135]. However, experiments in knockout mice for several tight junction proteins revealed that those animals developed only mild lung phenotypes but they showed an increased susceptibility for lung injury [64, 75]. Besides barrier function, decreased fluid resorption across the airway epithelium is an additional susceptibility factor for lung injury and respiratory distress syndrome and worsens the clinical outcome in patients [136]. Airway and alveolar epithelia compensates for alveolar oedema formation or elevated apical surface liquid volumes by up-regulating active ion resorption [31, 39, 49, 55, 92, 123] or via increasing transcellular water permeability [110] to facilitate fluid clearance.

However, the permselectivity of TJ is considered as a prerequisite for a resorptive fluid transport across epithelia [76], and indeed, TJ perturbation by cldn4 down-regulation is associated with a decreased alveolar fluid clearance [105]. Given this evidence, it is conclusive that an increase of transepithelial transport could not sufficiently compensate lung oedema formation in the presence of TJ damage. Indeed, up-regulation of transepithelial transport capacity alone did not sufficiently compensate exudate formation in a mouse model of LPS-induced lung injury. Instead, exudate clearance and lung symptoms significantly improve, when transepithelial transport capacity was increased in combination with a restoration of TJ tightness [81]. Therefore, elucidating mechanisms of TJ breakdown during lung inflammation or identifying protective mechanisms that prevent inflammatory TJ damage will help to prevent lung injury or respiratory distress syndrome in patients with lung inflammation.

## Concluding remarks

Inflammatory lung diseases constitute a broad spectrum of diseases. They are a major risk for life-threatening lung injury and respiratory distress syndrome [135]. The up-to-date therapeutic approaches focus on resolving inflammation.

Formation of lung oedema and exudates causes respiratory distress, and more recent studies demonstrated that TJ damage does not only cause exudate formation but also attenuates its clearance [81, 105]. Therapeutic strategies that attenuate TJ damage during inflammation and/or support TJ restoration will improve clinical outcome of patients. Elaborating the function of TJ and their molecular regulation in the lung will enhance our understanding of the lung epithelium itself and will help to develop novel strategies to treat patients with inflammatory lung diseases.

## References

[1] Abe A, Takano K, Kojima T, Nomura K, Kakuki T, Kaneko Y, Yamamoto M, Takahashi H, Himi T (2016). Interferon-gamma increased epithelial barrier function via upregulating claudin-7 expression in human submandibular gland duct epithelium. J Mol Histol.

[2] Ablimit A, Hasan B, Lu W, Qin W, Wushouer Q, Zhong N, Upur H (2013). Changes in water channel aquaporin 1 and aquaporin 5 in the small airways and the alveoli in a rat asthma model. Micron.

[3] Abramson MJ, Perret JL, Dharmage SC, McDonald VM, McDonald CF (2014). Distinguishing adult-onset asthma from COPD: a review and a new approach. Int J COPD.

[4] Akaiwa M, Yu B, Umeshita-Suyama R, Terada N, Suto H, Koga T, Arima K, Matsushita S, Saito H, Ogawa H, Furue M, Hamasaki N, Ohshima K, Izuhara K (2001). Localization of human interleukin 13 receptor in non-Haematopoietic cells. Cytokine.

[5] Al-Sadi R, Guo S, Ye D, Rawat M, Ma TY (2016). TNF-?? Modulation of intestinal tight junction permeability is mediated by NIK/IKK-β Axis activation of the canonical NF-??B pathway. Am J Pathol.

[6] Alexandre MD, Lu Q, Chen Y-H (2005). Overexpression of claudin-7 decreases the paracellular Cl- conductance and increases the paracellular Na^+^ conductance in LLC-PK1 cells. J Cell Sci.

[7] Amasheh M, Fromm A, Krug SM, Amasheh S, Andres S, Zeitz M, Fromm M, Schulzke J-D (2010). TNFalpha-induced and berberine-antagonized tight junction barrier impairment via tyrosine kinase Akt and NFκB signaling. J Cell Sci.

[8] Amasheh S, Meiri N, Gitter AH, Schöneberg T, Mankertz J, Schulzke JD, Fromm M (2002). Claudin-2 expression induces cation-selective channels in tight junctions of epithelial cells. J Cell Sci.

[9] Angelow S, Kim K-J, Yu ASL (2006). Claudin-8 modulates paracellular permeability to acidic and basic ions in MDCK II cells. J Physiol.

[10] Armstrong SM, Wang C, Tigdi J, Si X, Dumpit C, Charles S, Gamage A, Moraes TJ, Lee WL (2012) Influenza infects lung microvascular endothelium leading to microvascular leak: role of apoptosis and claudin-5. PLoS One 7 10.1371/journal.pone.0047323 PMC3480371 23115643

[11] Aslam M, Ahmad N, Srivastava R, Hemmer B (2012). TNF-α induced NFκB signaling and p65 (RelA) overexpression repress Cldn5 promoter in mouse brain endothelial cells. Cytokine.

[12] Asokananthan N, Graham PT, Fink J, Knight DA, Bakker AJ, McWilliam AS, Thompson PJ, Stewart GA (2002). Activation of protease-activated receptor (PAR)-1, PAR-2, and PAR-4 stimulates IL-6, IL-8, and prostaglandin E2 release from human respiratory epithelial cells. J Immunol.

[13] Aveleira A, Lin C, Abcouwer SF, Ambro F (2010). TNF-α signals through PKCz/NF-kB to Alter the tight junction complex and increase retinal endothelial cell permeability. Diabetes.

[14] Barnes PJ (2009). The cytokine network in chronic obstructive pulmonary disease. Am J Respir Cell Mol Biol.

[15] Beale J, Jayaraman A, Jackson DJ, Macintyre JDR, Edwards MR, Walton RP, Zhu J, Man Ching Y, Shamji B, Edwards M, Westwick J, Cousins DJ, Yi Hwang Y, McKenzie A, Johnston SL, Bartlett NW (2014). Rhinovirus induced IL-25 in asthma exacerbation drives type-2 immunity and allergic pulmonary inflammation Europe PMC funders group. Sci Transl Med Oct.

[16] Den Beste KA, Hoddeson EK, Parkos CA, Nusrat A, Wise SK (2013). Epithelial permeability alterations in an in vitro air-liquid interface model of allergic fungal rhinosinusitis. Int. Forum Allergy Rhinol..

[17] Beutheu Youmba S, Belmonte L, Galas L, Boukhettala N, Bôle-Feysot C, Déchelotte P, Coëffier M (2012). Methotrexate modulates tight junctions through NF-κB, MEK, and JNK pathways. J Pediatr Gastroenterol Nutr.

[18] Burek M, Förster CY (2009). Cloning and characterization of the murine claudin-5 promoter. Mol Cell Endocrinol.

[19] Chao Y-C, Pan S-H, Yang S-C, Yu S-L, Che T-F, Lin C-W, Tsai M-S, Chang G-C, Wu C-H, Wu Y-Y, Lee Y-C, Hong T-M, Yang P-C (2009). Claudin-1 is a metastasis suppressor and correlates with clinical outcome in lung adenocarcinoma. Am J Respir Crit Care Med.

[20] Chiba T, Kanda A, Ueki S, Ito W, Kamada Y, Oyamada H, Saito N, Kayaba H, Chihara J (2006). Prostaglandin D2 induces IL-8 and GM-CSF by bronchial epithelial cells in a CRTH2-independent pathway. Int Arch Allergy Immunol.

[21] Chow AW, Ko WH, Liang JF Wong JS Chong FUY, Tang NL. Polarized secretion of interleukin (IL)-6 and IL-8 by human airway epithelia 16HBE14o- cells in response to cationic polypeptide challenge. PLoS One 5, 2010 10.1371/journal.pone.0012091 PMC2920803 20711426

[22] Cong X, Zhang Y, Li J, Mei M, Ding C, Xiang R-L, Zhang L-W, Wang Y, Wu L-L, Yu G-Y (2015). Claudin-4 is required for modulation of paracellular permeability by muscarinic acetylcholine receptor in epithelial cells. J Cell Sci.

[23] Corrigan CJ, Wang W, Meng Q, Fang C, Eid G, Caballero MR, Lv Z, An Y, Wang YH, Liu YJ, Kay AB, Lee TH, Ying S (2011). Allergen-induced expression of IL-25 and IL-25 receptor in atopic asthmatic airways and late-phase cutaneous responses. J. Allergy Clin. Immunol..

[24] Coyne CB, Gambling TM, Boucher RC, Carson JL, Johnson LG (2003). Role of claudin interactions in airway tight junctional permeability. Am. J. Physiol. Lung Cell. Mol. Physiol..

[25] Coyne CB, Vanhook MK, Gambling TM, Carson JL, Boucher RC, Johnson LG (2002). Regulation of airway tight junctions by proinflammatory cytokines. Mol Biol Cell.

[26] Crapo JD, Young SL, Fram EK, Pinkerton KE, Barry BE, Crapo RO (1983). Morphometric characteristics of cells in the alveolar region of mammalian lungs. Am Rev Respir Dis.

[27] Cromwell O, Hamid Q, Corrigan CJ, Barkans J, Meng Q, Collins PD (1992). **Kay a B**. Expression and generation of interleukin-8, IL-6 and granulocyte-macrophage colony-stimulating factor by bronchial epithelial cells and enhancement by IL-1 beta and tumour necrosis factor-alpha. Immunology.

[28] Daugherty BL, Mateescu M, Patel AS, Wade K, Kimura S, Gonzales LW, Guttentag S, Ballard PL, Koval M (2004). Developmental regulation of claudin localization by fetal alveolar epithelial cells. Am. J. Physiol. Lung Cell. Mol. Physiol..

[29] Day SB, Ledford JR, Zhou P, Lewkowich IP, Page K (2011). German cockroach proteases and protease-activated receptor-2 regulate chemokine production and dendritic cell recruitment. J Innate Immun.

[30] Ding L, Lu Z, Foreman O, Tatum R, Lu Q, Renegar R, Cao J, Chen Y (2012). Inflammation and disruption of the mucosal architecture in claudin-7-deficient mice. Gastroenterology.

[31] Donaldson SH, Hirsh A, Li DC, Holloway G, Chao J, Boucher RC, Gabriel SE (2002). Regulation of the epithelial sodium channel by serine proteases in human airways. J Biol Chem.

[32] Dong C, Wang G, Li B, Xiao K, Ma Z, Huang H, Wang X, Bai C (2012). Anti-asthmatic agents alleviate pulmonary edema by upregulating AQP1 and AQP5 expression in the lungs of mice with OVA-induced asthma. Respir Physiol Neurobiol.

[33] Farias R, Rousseau S (2015). The TAK1 → IKKβ → TPL2 → MKK1/MKK2 signaling Cascade regulates IL-33 expression in cystic fibrosis airway epithelial cells following infection by Pseudomonas Aeruginosa. *Front. cell*. Dev Biol.

[34] Fernandez AL, Koval M, Fan X, Guidot DM (2007). Chronic alcohol ingestion alters claudin expression in the alveolar epithelium of rats. Alcohol.

[35] Folkesson HG, Matthay MA, Hasegawa H, Kheradmand F, Verkman AS (1994). Transcellular water transport in lung alveolar epithelium through mercury-sensitive water channels. [online]. Proc Natl Acad Sci U S A.

[36] Frank JA (2012). Claudins and alveolar epithelial barrier function in the lung. Ann N Y Acad Sci.

[37] Fujibe M, Chiba H, Kojima T, Soma T, Wada T, Yamashita T, Sawada N (2004). Thr203 of claudin-1, a putative phosphorylation site for MAP kinase, is required to promote the barrier function of tight junctions. Exp Cell Res.

[38] Furuse M, Furuse K, Sasaki H, Tsukita S (2001). Conversion of zonulae occludentes from tight to leaky strand type by introducing claudin-2 into Madin-Darby canine kidney I cells. J Cell Biol.

[39] Garcia-Caballero A, Rasmussen JE, Gaillard E, Watson MJ, Olsen JC, Donaldson SH, Stutts MJ, Tarran R (2009). SPLUNC1 regulates airway surface liquid volume by protecting ENaC from proteolytic cleavage. Proc Natl Acad Sci U S A.

[40] Ghosh S, May MJ, Kopp EB (1998). NF- κ B AND REL PROTEINS : evolutionarily conserved mediators of immune responses. Annu Rev iImunology.

[41] Guidot DMBE, Modelska K, Lois MB, Jain L, Moss IM, Pittet J-F, LAS B (2000). Ethanol ingestion via glutathione depletion impairs alveolar epithelial barrier function in rats [online]. Am J Physiol - Lung Cell Mol Physiol.

[42] Günzel D, Yu ASL. Claudins and the modulation of tight junction permeability. . 10.1152/physrev.00019.2012 PMC3768107 23589827

[43] Hardyman MA, Wilkinson E, Martin E, Jayasekera NP, Blume C, Swindle EJ, Gozzard N, Holgate ST, Howarth PH, Davies DE, Collins JE (2013). TNF-α-mediated bronchial barrier disruption and regulation by src-family kinase activation. J Allergy Clin Immunol.

[44] Hashimoto K, Oshima T, Tomita T, Kim Y, Matsumoto T, Joh T, Miwa H (2008). Oxidative stress induces gastric epithelial permeability through claudin-3. Biochem Biophys Res Commun.

[45] Haven N, Biology C (1988). Cells which differ in transepithelial resistance. Cell.

[46] Heinzmann A, Mao XQ, Akaiwa M, Kreomer RT, Gao PS, Ohshima K, Umeshita R, Abe Y, Braun S, Yamashita T, Roberts MH, Sugimoto R, Arima K, Arinobu Y, Yu B, Kruse S, Enomoto T, Dake Y, Kawai M, Shimazu S, Sasaki S, Adra CN, Kitaichi M, Inoue H, Yamauchi K, Tomichi N, Kurimoto F, Hamasaki N, Hopkin JM, Izuhara K, Shirakawa T, Deichmann KA (2000). Genetic variants of IL-13 signalling and human asthma and atopy. Hum Mol Genet.

[47] Hellermann GR, Nagy SB, Kong X, Lockey RF, Mohapatra SS (2002). Mechanism of cigarette smoke condensate-induced acute inflammatory response in human bronchial epithelial cells. Respir Res.

[48] Heyen L, Müller U, Siegemund S, Schulze B, Protschka M, Alber G, Piehler D. Lung epithelium is the major source of IL-33 and is regulated by IL-33-dependent and IL-33-independent mechanisms in pulmonary cryptococcosis. *Pathog. Dis.* 74: ftw086, 2016. 10.1093/femspd/ftw086 27596810

[49] Hobbs CA, Blanchard MG, Alijevic O, Tan CDA, Kellenberger S, Bencharit S, Cao R, Kesimer M, Walton WG, Henderson AG, Redinbo MR, Stutts MJ, Tarran R (2013). Identification of the SPLUNC1 ENaC-inhibitory domain yields novel strategies to treat sodium hyperabsorption in cystic fibrosis airway epithelial cultures. Am. J. Physiol. Lung Cell. Mol. Physiol..

[50] Holter JF, Weiland JE, Pacht ER, Gadek JE, Davis WB (1986). Protein permeability in the adult respiratory distress syndrome loss of size selectivity of the alveolar epithelium. J Clin Invest.

[51] Hou J, Gomes AS, Paul DL, Goodenough DA (2006). Study of claudin function by RNA interference. J Biol Chem.

[52] Hou J, Renigunta A, Yang J, Waldegger S (2010). Claudin-4 forms paracellular chloride channel in the kidney and requires claudin-8 for tight junction localization. Proc Natl Acad Sci U S A.

[53] Hristova M, Habibovic A, Veith C, Janssen-Heininger YMW, Dixon AE, Geiszt M, Van Der Vliet A (2016). Airway epithelial dual oxidase 1 mediates allergen-induced IL-33 secretion and activation of type 2 immune responses. J. Allergy Clin. Immunol..

[54] Huang LY, Stuart C, Takeda K, D’Agnillo F, Golding B, Morita K, Sasaki H, Furuse M, Tsukita S, Opal S, van der Poll T, Goddard L, Iruela-Arispe M, Armstrong S, Wang C, Tigdi J, Si X, Dumpit C, Charles S, Armstrong S, Darwish I, Lee W, Vervaeke P, Vermeire K, Liekens S, D’Agnillo F, Williams M, Moayeri M, Warfel J, Dalrymple N, Mackow E, Lee N, Wong C, Hui D, Chan P, Kawai T, Akira S, Kumar H, Kawai T, Akira S, Aoshi T, Koyama S, Kobiyama K, Akira S, Ishii K, Chattopadhyay S, Sen G, Stowell N, Seideman J, Raymond H, Smalley K, Lamb R, Egenolf D, Zeng H, Pappas C, Belser J, Houser K, Zhong W, Wadford D, Patel M, Shirey K, Pletneva L, Boukhvalova M, Garzino-Demo A, Vogel S, Balint Z, Zabini D, Konya V, Nagaraj C, Vegh A, Varo G, Rezaee F, Meednu N, Emo J, Saatian B, Chapman T, Naydenov N, Livak K, Schmittgen T, Ritter M, Mennerich D, Weith A, Seither P, Kuznik A, Bencina M, Svajger U, Jeras M, Rozman B, Jerala R, Mazzon E, Cuzzocrea S, Capaldo C, Nusrat A, Clark P, Kim R, Pober J, Kluger M, Alexopoulou L, Holt A, Medzhitov R, Flavell R, Lundberg A, Drexler S, Monaco C, Williams L, Sacre S, Feldmann M, Slater L, Bartlett N, Haas J, Zhu J, Message S, Walton R, Chalmers A, Whitley P, Chiang E, Persaud-Sawin D, Kulkarni S, Garcia J, Imani F, Kasa A, Csortos C, Verin A, Majde J, Brown R, Jones M, Dieffenbach C, Maitra N, Krueger J (2016) Poly(I:C) induces human lung endothelial barrier dysfunction by disrupting tight junction expression of claudin-5. PLoS One 11:–e0160875 10.1371/journal.pone.0160875 PMC4978501 27504984

[55] Hughey RP, Bruns JB, Kinlough CL, Harkleroad KL, Tong Q, Carattino MD, Johnson JP, Stockand JD, Kleyman TR (2004). Epithelial sodium channels are activated by furin-dependent proteolysis. J Biol Chem.

[56] Inai T, Kobayashi J, Shibata Y (1999). Claudin-1 contributes to the epithelial barrier function in MDCK cells. Eur J Cell Biol.

[57] Van Itallie CM, Fanning AS, Anderson JM (2003). Reversal of charge selectivity in cation or anion-selective epithelial lines by expression of different claudins. Am. J. Physiol. Renal Physiol..

[58] Jairaman A, Maguire CH, Schleimer RP, Prakriya M (2016). Allergens stimulate store-operated calcium entry and cytokine production in airway epithelial cells. Nat Publ Gr.

[59] Jairaman A, Yamashita M, Schleimer RP, Prakriya M (2015). Store-operated Ca^2+^ release-activated Ca^2+^ channels regulate PAR2-activated Ca^2+^ signaling and cytokine production in airway epithelial cells. J Immunol.

[60] Jeansonne B, Lu Q, Goodenough DA, Chen YH (2003). Claudin-8 interacts with multi-PDZ domain protein 1 (MUPP1) and reduces paracellular conductance in epithelial cells. [online]. Cell Mol Biol (Noisy-le-grand).

[61] Jin W, Rong L, Liu Y, Song Y, Li Y, Pan J (2013). Increased claudin-3, −4 and −18 levels in bronchoalveolar lavage fluid reflect severity of acute lung injury. Respirology.

[62] Kaarteenaho-Wiik R, Soini Y (2009). Claudin-1, −2, −3, −4, −5, and −7 in usual interstitial pneumonia and sarcoidosis. J Histochem Cytochem.

[63] Kaarteenaho R, Merikallio H, Lehtonen S, Harju T, Soini Y (2010). Divergent expression of claudin −1, −3, −4, −5 and −7 in developing human lung. Respir Res.

[64] Kage H, Flodby P, Gao D, Kim YH, Marconett CN, DeMaio L, Kim K-J, Crandall ED, Borok Z (2014). Claudin 4 knockout mice: normal physiological phenotype with increased susceptibility to lung injury. Am. J. Physiol. Lung Cell. Mol. Physiol..

[65] Khosravi AR, Erle DJ (2016). Chitin-induced airway epithelial cell innate immune responses are inhibited by Carvacrol/thymol. PLoS One.

[66] Kielgast F, Schmidt H, Braubach P, Winkelmann VE, Thompson KE, Frick M, Dietl P, Wittekindt OH (2016). Glucocorticoids regulate tight junction permeability of lung epithelia by modulating claudin 8. Am J Respir Cell Mol Biol.

[67] Kim Y-M, Reed W, Wu W, Bromberg PA, Graves LM, Samet JM (2006). Zn^2+^-induced IL-8 expression involves AP-1, JNK, and ERK activities in human airway epithelial cells. Am J Physiol Lung Cell Mol Physiol.

[68] King C, Brennan S, Thompson PJ, Stewart GA, King C, Brennan S, Thompson PJ, Stewart GA. Dust Mite Proteolytic Allergens Induce Cytokine Release from Cultured Airway Epithelium.. 9759888

[69] Kotsimbos TC, Ghaffar O, Minshall EM, Humbert M, Durham SR, Pfister R, Menz G, Kay AB, Hamid QA (1998). Expression of the IL-4 receptor alpha-subunit is increased in bronchial biopsy specimens from atopic and nonatopic asthmatic subjects. [online]. J. Allergy Clin. Immunol.

[70] Koval M (2009). Tight junctions, but not too tight: fine control of lung permeability by claudins. AmJ Physiol Lung Cell Mol Physiol.

[71] Koval M (2013). Claudin heterogeneity and control of lung tight junctions. Annu Rev Physiol.

[72] Krause G, Winkler L, Mueller SL, Haseloff RF, Piontek J, Blasig IE (2008). Structure and function of claudins. Biochim. Biophys. Acta-Biomembr..

[73] Ladwein M, Pape UF, Schmidt DS, Schnölzer M, Fiedler S, Langbein L, Franke WW, Moldenhauer G, Zöller M (2005). The cell-cell adhesion molecule EpCAM interacts directly with the tight junction protein claudin-7. Exp Cell Res.

[74] Lafemina MJ, Rokkam D, Chandrasena A, Pan J, Bajaj A, Johnson M, Frank JA (2010). Keratinocyte growth factor enhances barrier function without altering claudin expression in primary alveolar epithelial cells. AJP Lung Cell Mol Physiol.

[75] LaFemina MJ, Sutherland KM, Bentley T, Gonzales LW, Allen L, Chapin CJ, Rokkam D, Sweerus KA, Dobbs LG, Ballard PL, Frank JA (2014). Claudin-18 deficiency results in alveolar barrier dysfunction and impaired alveologenesis in mice. Am J Respir Cell Mol Biol.

[76] Larsen EH, Willumsen NJ, Møbjerg N, Sørensen JN (2009). The lateral intercellular space as osmotic coupling compartment in isotonic transport. Acta Physiol (Oxf).

[77] Li B, Dong C, Wang G, Zheng H, Wang X, Bai C (2011). Pulmonary epithelial CCR3 promotes LPS-induced lung inflammation by mediating release of IL-8. J Cell Physiol.

[78] Li G, Flodby P, Luo J, Kage H, Sipos A, Gao D, Ji Y, Beard LML, Marconett CN, DeMaio L, Kim YH, Kim KJ, Laird-Offringa IA, Minoo P, Liebler JM, Zhou B, Crandall ED, Borok Z (2014). Knockout mice reveal key roles for claudin 18 in alveolar barrier properties and fluid homeostasis. Am J Respir Cell Mol Biol.

[79] Li H, Singh S, Potula R, Persidsky Y, Kanmogne GD (2014). Dysregulation of claudin-5 in HIV-induced interstitial pneumonitis and lung vascular injury: protective role of peroxisome proliferator-activated receptor-gamma. Am J Respir Crit Care Med.

[80] Licona-Limón P, Kim LK, Palm NW, Flavell R (2013). **A**. TH2, allergy and group 2 innate lymphoid cells. Nat Immunol.

[81] Lin X, Barravecchia M, Kothari P, Young JL, Dean DA. beta1-NaK-ATPase gene therapy upregulates tight junctions to rescue lipopolysaccharide-induced acute lung injury. Gene Ther*.*: 489–499, 2016. 10.1038/gt.2016.19 26910760

[82] Lu Z, Kim DH, Fan J, Lu Q, Verbanac K, Ding L, Renegar R, Chen Y-H (2015). A non-tight junction function of claudin-7-interaction with integrin signaling in suppressing lung cancer cell proliferation and detachment. Mol Cancer.

[83] Ma T, Fukuda N, Song Y, Matthay MA, Verkman AS (2000). Lung fluid transport in aquaporin-5 knockout mice. J Clin Invest.

[84] Maclaren OJ, Sneyd J, Crampin EJ (2013). What do aquaporin knockout studies tell us about fluid transport in epithelia?. J Membr Biol.

[85] Masaki T, Kojima T, Okabayashi T, Ogasawara N, Ohkuni T, Obata K, Takasawa A, Murata M, Tanaka S, Hirakawa S, Fuchimoto J, Ninomiya T, Fujii N, Tsutsumi H, Himi T, Sawada N (2011). A nuclear factor-κB signaling pathway via protein kinase C δ regulates replication of respiratory syncytial virus in polarized normal human nasal epithelial cells. Mol Biol Cell.

[86] Mazzon E, Cuzzocrea S (2007). Role of TNF-α in lung tight junction alteration in mouse model of acute lung inflammation. Respir Res.

[87] McCarthy KM, Francis SA, McCormack JM, Lai J, Rogers RA, Skare IB, Lynch RD, Schneeberger EE (2000). Inducible expression of claudin-1-myc but not occludin-VSV-G results in aberrant tight junction strand formation in MDCK cells. J Cell Sci.

[88] Mei M, Xiang RL, Cong X, Zhang Y, Li J, Yi X, Park K, Han JY, Wu LL, Yu GY (2015). Claudin-3 is required for modulation of paracellular permeability by TNF-α through ERK1/2/slug signaling axis in submandibular gland. Cell Signal.

[89] Michikawa H, Fujita-Yoshigaki J, Sugiya H (2008). Enhancement of barrier function by overexpression of claudin-4 in tight junctions of submandibular gland cells. Cell Tissue Res.

[90] Milatz S, Krug SM, Rosenthal R, Günzel D, Müller D, Schulzke JD, Amasheh S, Fromm M (2010). Claudin-3 acts as a sealing component of the tight junction for ions of either charge and uncharged solutes. Biochim Biophys Acta-Biomembr.

[91] Mitchell LA, Overgaard CE, Ward C, Margulies SS, Koval M, La M, Ce O, Ward C, Ss M, Koval M (2011). Differential effects of claudin-3 and claudin-4 on alveolar epithelial barrier function.

[92] Myerburg MM, Harvey PR, Heidrich EM, Pilewski JM, Butterworth MB (2010). Acute regulation of the epithelial sodium channel in airway epithelia by proteases and trafficking. Am J Respir Cell Mol Biol.

[93] Nabe T, Wakamori H, Yano C, Nishiguchi A, Yuasa R, Kido H, Tomiyama Y, Tomoda A, Kida H, Takiguchi A, Matsuda M, Ishihara K, Akiba S, Ohya S, Fukui H, Mizutani N, Yoshino S (2015). Production of interleukin (IL)-33 in the lungs during multiple antigen challenge-induced airway inflammation in mice, and its modulation by a glucocorticoid. Eur J Pharmacol.

[94] Niimi T, Nagashima K, Ward J (2001). 18, a novel downstream target gene for the T/EBP/NKX2. 1 homeodomain transcription factor, encodes lung-and stomach-specific isoforms through alternative splicing. Cell Biol.

[95] Nomura K, Obata K, Keira T, Miyata R, Hirakawa S, Takano K, Kohno T, Sawada N, Himi T, Kojima T (2014). Pseudomonas Aeruginosa elastase causes transient disruption of tight junctions and downregulation of PAR-2 in human nasal epithelial cells. Respir Res.

[96] Ogasawara N, Kojima T, Go M, Ohkuni T, Koizumi JI, Kamekura R, Masaki T, Murata M, Tanaka S, Fuchimoto J, Himi T, Sawada N (2010). PPARγ agonists upregulate the barrier function of tight junctions via a PKC pathway in human nasal epithelial cells. Pharmacol Res.

[97] Oh K, Seo MW, Lee GY, Byoun O-J, Kang H-R, Cho S-H, Lee D-S (2013). Airway epithelial cells initiate the allergen response through transglutaminase 2 by inducing IL-33 expression and a subsequent Th2 response. Respir Res.

[98] Österlund C, Grönlund H, Polovic N, Sundström S, Gafvelin G, Bucht A (2009). The non-proteolytic house dust mite allergen der p 2 induce NF-κB and MAPK dependent activation of bronchial epithelial cells. Clin Exp Allergy.

[99] Pasparakis M (2009). Regulation of tissue homeostasis by NF-κB signalling: implications for inflammatory diseases. Nat Rev Immunol.

[100] Petecchia L, Sabatini F, Usai C, Caci E, Varesio L, Rossi GA (2012). Cytokines induce tight junction disassembly in airway cells via an EGFR-dependent MAPK/ERK1/2-pathway. Lab Investig.

[101] Piper SC, Ferguson J, Kay L, Parker LC, Sabroe I, Sleeman MA, Briend E, Finch DK. The Role of Interleukin-1 and Interleukin-18 in Pro-Inflammatory and Anti-Viral Responses to Rhinovirus in Primary Bronchial Epithelial Cells. PLoS One 8, 2013. 10.1371/journal.pone.0063365 PMC3665753 23723976

[102] Préfontaine D, Nadigel J, Chouiali F, Audusseau S, Semlali A, Chakir J, Martin JG, Hamid Q (2010). Increased IL-33 expression by epithelial cells in bronchial asthma. J. Allergy Clin. Immunol..

[103] Profita M, Bonanno A, Siena L, Ferraro M, Montalbano AM, Pompeo F, Riccobono L, Pieper MP, Gjomarkaj M (2008). Acetylcholine mediates the release of IL-8 in human bronchial epithelial cells by a NFkB/ERK-dependent mechanism. Eur J Pharmacol.

[104] Ritchie AI, Jackson DJ, Edwards MR, Johnston SL (2016). Airway epithelial orchestration of innate immune function in response to virus infection: a focus on asthma. Ann Am Thorac Soc.

[105] Rokkam D, Lafemina MJ, Lee JW, Matthay MA, Frank JA (2011). Claudin-4 levels are associated with intact alveolar fluid clearance in human lungs. Am J Pathol.

[106] Rosenthal R, Milatz S, Krug SM, Oelrich B, Schulzke J-D, Amasheh S, Günzel D, Fromm M (2010). Claudin-2, a component of the tight junction, forms a paracellular water channel. J Cell Sci.

[107] Saatian B, Rezaee F, Desando S, Emo J, Chapman T, Knowlden S, Georas SN (2013). Interleukin-4 and interleukin-13 cause barrier dysfunction in human airway epithelial cells. Tissue barriers.

[108] Sabnis AS, Reilly CA, Veranth JM, Ost GS (2008). Increased transcription of cytokine genes in human lung epithelial cells through activation of a TRPM8 variant by cold temperatures. Am. J. Physiol. Lung Cell. Mol. Physiol.

[109] Schlingmann B, Molina SA, Koval M (2015). Claudins: gatekeepers of lung epithelial function. Semin Cell Dev Biol.

[110] Schmidt H, Michel C, Braubach P, Fauler M, Neubauer D, Thompson KE, Frick M, Mizaikoff B, Dietl P, Wittekindt OH (2016). Water permeability adjusts resorption in lung epithelia to increased apical surface liquid volumes. Am J Respir Cell Mol Biol.

[111] Shiozaki A, Bai X, Shen-Tu G, Moodley S, Takeshita H, Fung S-Y, Wang Y, Keshavjee S, Liu M (2012). Claudin 1 mediates TNFα-induced gene expression and cell migration in human lung carcinoma cells. PLoS One.

[112] Simet SM, Wyatt TA, Devasure J, Yanov D, Allen-Gipson D, Sisson JH (2012). Alcohol increases the permeability of airway epithelial tight junctions in Beas-2B and NHBE cells. Alcohol Clin Exp Res.

[113] Singha O, Kengkoom K, Chaimongkolnukul K, Cherdyu S, Pongponratn E, Ketjareon T, Panavechkijkul Y, Ampawong S (2013). Pulmonary edema due to oral gavage in a toxicological study related to aquaporin-1, −4 and −5 expression. J Toxicol Pathol.

[114] Song Y, Jayaraman S, Yang B, Matthay MA, Verkman AS (2001). Role of aquaporin water channels in airway fluid transport, humidification, and surface liquid hydration. [online]. J Gen Physiol.

[115] Standiford TJ, Kunkel SL, Basha MA, Chensue SW, Iii JPL, Toews GB, Westwick J, Strieter RM (1990). Lnterleukin-8 Gene expression by a pulmonary epithelial. Cell Line.

[116] Stokes JR, Casale TB (2016). Characterization of asthma endotypes: implications for therapy. Ann Allergy, Asthma Immunol.

[117] Sun Y, Wu F, Sun F, Huang P (2008). Adenosine promotes IL-6 release in airway epithelia. J Immunol.

[118] Sweerus K, Lachowicz-Scroggins M, Gordon E, LaFemina M, Huang X, Parikh M, Kanegai C, Fahy JV, Frank JA (2016). Claudin-18 deficiency is associated with airway epithelial barrier dysfunction and asthma. J Allergy Clin Immunol.

[119] Takizawa H, Ohtoshi T, Kawasaki S, Abe S, Sugawara I, Nakahara K, Matsushima K, Kudoh S (2000). Diesel exhaust particles activate human bronchial epithelial cells to express inflammatory mediators in the airways: a review. Respirology.

[120] Takizawa H, Ohtoshi T, Kikutani T, Okazaki H, Akiyama N, Sato M, Shoji S, Ito K (1995). Histamine activates bronchial epithelial cells to release inflammatory cytokines in vitro. [online]. Int Arch Allergy Immunol.

[121] Tal TL, Simmons SO, Silbajoris R, Dailey L, Cho SH, Ramabhadran R, Linak W, Reed W, Bromberg PA, Samet JM (2010). Differential transcriptional regulation of IL-8 expression by human airway epithelial cells exposed to diesel exhaust particles. Toxicol Appl Pharmacol.

[122] Tanaka H, Takechi M, Kiyonari H, Shioi G, Tamura A, Tsukita S (2015). Intestinal deletion of claudin-7 enhances paracellular organic solute flux and initiates colonic inflammation in mice. Gut.

[123] Tarran R, Trout L, Donaldson SH, Boucher RC (2006). Soluble mediators, not cilia, determine airway surface liquid volume in normal and cystic fibrosis superficial airway epithelia. J. Gen. Physiol..

[124] Tatum R, Zhang Y, Lu Q, Kim K, Jeansonne BG, Chen YH (2007). WNK4 phosphorylates ser206 of claudin-7 and promotes paracellular Cl- permeability. FEBS Lett.

[125] Tatum R, Zhang Y, Salleng K, Lu Z, Lin J-J, Lu Q, Jeansonne BG, Ding L, Chen Y-H (2010). Renal salt wasting and chronic dehydration in claudin-7-deficient mice. Am J Physiol Renal Physiol.

[126] Tokumasu R, Yamaga K, Yamazaki Y, Murota H, Suzuki K, Tamura A, Bando K, Furuta Y, Katayama I, Tsukita S (2016). Dose-dependent role of claudin-1 in vivo in orchestrating features of atopic dermatitis. Proc Natl Acad Sci U S A.

[127] Towne JE, Harrod KS, Krane CM, Menon AG (2000). Decreased expression of aquaporin (AQP)1 and AQP5 in mouse lung after acute viral infection. Am J Respir Cell Mol Biol.

[128] Usmani SM, von Einem J, Frick M, Miklavc P, Mayenburg M, Husmann M, Dietl P, Wittekindt OH (2012). Molecular basis of early epithelial response to streptococcal exotoxin: role of STIM1 and Orai1 proteins. Cell Microbiol.

[129] van der Velden VHJ, Naber BAE, Wierenga-Wolf AF, Debets R, Savelkoul HFJ, Overbeek SE, Hoogsteden HC, Versnel MA (1998). Interleukin 4 receptors on human bronchial epithelial cells an in vivo and in vitro analysis of expression and function. Cytokine.

[130] Verkman AS (2007). Role of aquaporins in lung liquid physiology. Respir Physiol Neurobiol.

[131] Verkman AS, Yang B, Song Y, Manley GT, Ma T (2000). Role of water channels in fluid transport studied by phenotype analysis of aquaporin knockout mice. [online]. Exp Physiol.

[132] Wang F, Daugherty B, Keise LL, Wei Z, Foley JP, Savani RC, Koval M (2003). Heterogeneity of claudin expression by alveolar epithelial cells. Am J Respir Cell Mol Biol.

[133] Wang IM, Lin H, Goldman SJ, Kobayashi M (2004). STAT-1 is activated by IL-4 and IL-13 in multiple cell types. Mol Immunol.

[134] Ward C, Schlingmann BL, Stecenko AA, Guidot DM, Koval M (2015). NF-κB inhibitors impair lung epithelial tight junctions in the absence of inflammation. Tissue barriers.

[135] Ware LB, Matthay MA (2000). The acute respiratory distress syndrome. N Engl J Med.

[136] Ware LB, Matthay MA (2001). Alveolar fluid clearance is impaired in the majority of patients with acute lung injury and the acute respiratory distress syndrome. Am J Respir Crit Care Med.

[137] Way EE, Chen K, Kolls JK (2013). Dysregulation in lung immunity - the protective and pathologic Th17 response in infection. Eur J Immunol.

[138] Weibel ER (2015). On the tricks alveolar epithelial cells play to make a good lung. Am J Respir Crit Care Med.

[139] White SR, Martin LD, Stern R, Laxman B, Marroquin BA, Sr W, Ld M, Stern R, Laxman B (2010). **Expres- MBA**. Expression of IL-4 / IL-13 receptors in differentiating human airway epithelial cells. Am J Physiol Lung Cell Mol Physiol.

[140] Williams AE, Chambers RC (2014). The mercurial nature of neutrophils: still an enigma in ARDS?. Am. J. Physiol. Lung Cell. Mol. Physiol..

[141] Wilmes A, Aschauer L, Limonciel A, Pfaller W, Jennings P (2014). Evidence for a role of claudin 2 as a proximal tubular stress responsive paracellular water channel. Toxicol Appl Pharmacol.

[142] Wise SK, Laury AM, Katz EH, Den Beste KA, Parkos CA, Nusrat A (2014). Interleukin-4 and interleukin-13 compromise the sinonasal epithelial barrier and perturb intercellular junction protein expression. Int Forum Allergy Rhinol.

[143] Wissel H, Schulz C, Koehne P, Richter E, Maass M, Rüdiger M (2005). Chlamydophila pneumoniae induces expression of toll-like receptor 4 and release of TNF-alpha and MIP-2 via an NF-kappaB pathway in rat type II pneumocytes. Respir Res.

[144] Wray C, Mao Y, Pan J, Chandrasena A, Piasta F, Frank JA (2009). Claudin-4 augments alveolar epithelial barrier function and is induced in acute lung injury. Am. J. Physiol. Lung Cell. Mol. Physiol..

[145] Xiang RL, Mei M, Cong X, Li J, Zhang Y, Ding C, Wu LL, Yu GY (2014). Claudin-4 is required for AMPK-modulated paracellular permeability in submandibular gland cells. J Mol Cell Biol.

[146] Xiao M, Zhu T, Zhang W, Wang T, Shen YC, Wan QF, Wen FQ (2014). Emodin ameliorates LPS-induced acute lung injury, involving the inactivation of NF-κB in mice. Int J Mol Sci.

[147] Yang S-R, Chida AS, Bauter MR, Shafiq N, Seweryniak K, Maggirwar SB, Kilty I, Rahman I (2006). Cigarette smoke induces proinflammatory cytokine release by activation of NF-kappaB and posttranslational modifications of histone deacetylase in macrophages. Am J Physiol Lung Cell Mol Physiol.

[148] Yang SR, Valvo S, Yao H, Kode A, Rajendrasozhan S, Edirisinghe I, Caito S, Adenuga D, Henry R, Fromm G, Maggirwar S, Li JD, Bulger M, Rahman I (2008). IKKβ causes chromatin modification on pro-inflammatory genes by cigarette smoke in mouse lung. Am J Respir Cell Mol Biol.

[149] Yu ASL, Cheng MH, Angelow S, Günzel D, Kanzawa SA, Schneeberger EE, Fromm M, Coalson RD (2009). Molecular basis for cation selectivity in claudin-2-based paracellular pores: identification of an electrostatic interaction site. J Gen Physiol.

[150] Yu ASL, Enck AH, Lencer WI, Schneeberger EE (2003). Claudin-8 expression in Madin-Darby canine kidney cells augments the paracellular barrier to cation permeation. J Biol Chem.

[151] Yuan W, Li L, Hu Y, Li W, Guo Z, Huang W (2016). Inhibition of acute lung injury by TNFR-fc through regulation of an inflammation- oxidative stress pathway. PLoS One.

[152] Zhu T, Wang DX, Zhang W, Liao XQ, Guan X, Bo H, Sun JY, Huang NW, He J, Zhang YK, Tong J, Li CY. Andrographolide Protects against LPS-Induced Acute Lung Injury by Inactivation of NF-??B. *PLoS One* 8, 2013 10.1371/journal.pone.0056407 PMC3578846 23437127

